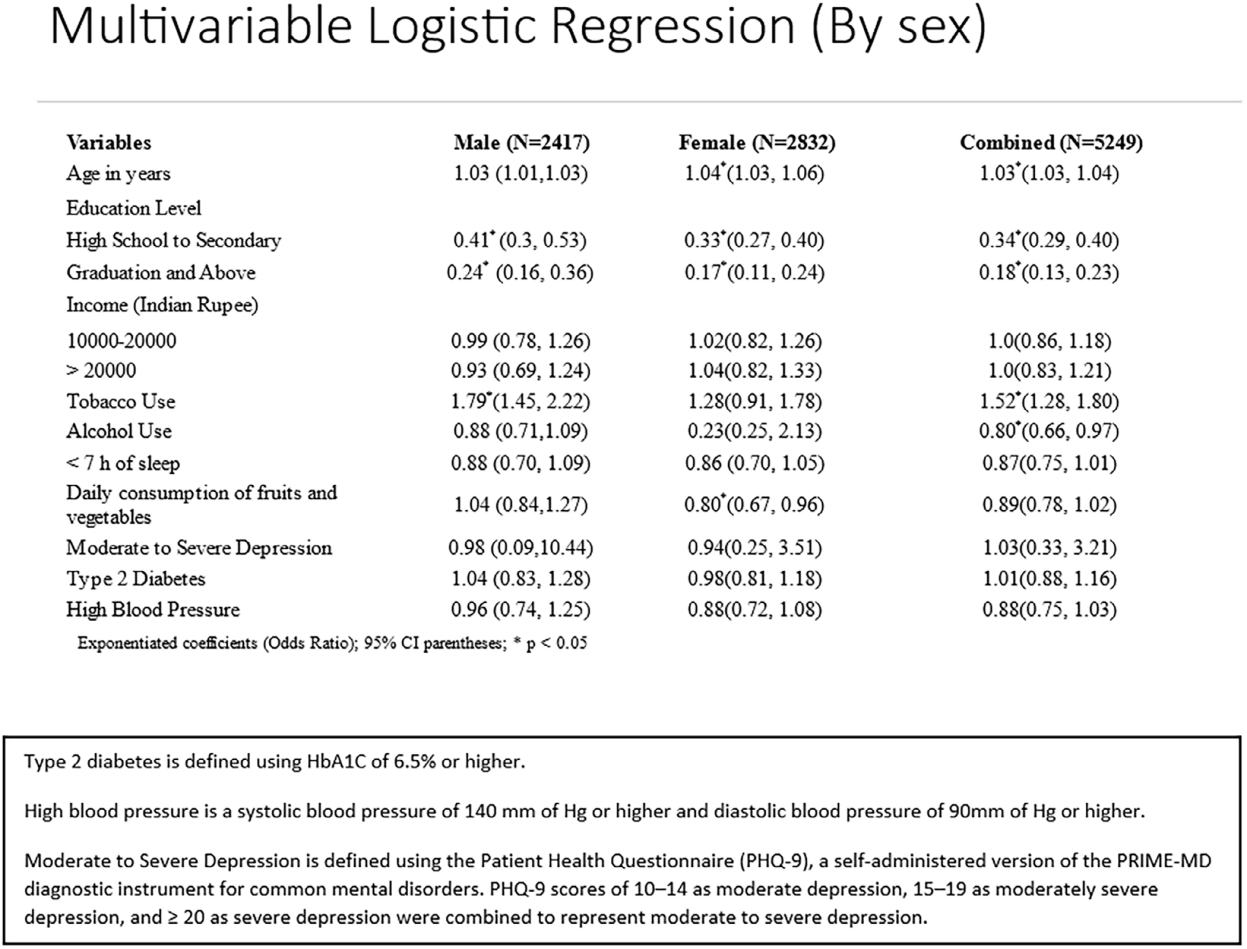# Cognitive Impairment and Its Risk Factors Among South Asians Living in India: Findings from the P‐CARRS Cohort Study

**DOI:** 10.1002/alz.088023

**Published:** 2025-01-09

**Authors:** Rima Pai, Dimple Kondal, Deepa Mohan, Rani Komal, Poongothai Subramani, Suvarna Alladi, Sailesh Mohan, Mohammed K Ali, Felicia Goldstein, Allan I. Levey, Nikhil Tandon, Dorairaj Prabhakaran, V Mohan, Venkat Narayan KM, Ram Jagannathan

**Affiliations:** ^1^ Hubert Department of Global Health, Rollins School of Public Healt, ATLANTA, GA USA; ^2^ Centre for Chronic Disease Control, New Delhi, Delhi India; ^3^ Madras Diabetes Research Foundation, Chennai, Tamil Nadu India; ^4^ National Institute of Mental Health and Neuro Sciences, Bengaluru, Karnataka India; ^5^ Hubert Department of Global Health, Rollins School of Public Health, Atlanta, GA USA; ^6^ Emory University School of Medicine, Atlanta, GA USA; ^7^ Emory University, Atlanta, GA USA; ^8^ All India Institute of Medical Sciences, New Delhi, Delhi India

## Abstract

**Background:**

To estimate the prevalence of cognitive impairment in a population‐based cohort from India and assess the potential modifiable factors of cognitive impairment.

**Method:**

We used the population‐representative data from the longitudinal Precision‐CARRS study from Delhi and Chennai, India. The cohort was recruited in two waves, CARRS‐1 in 2010‐11 and CARRS‐2 in 2015‐16. We administered the Mini‐Cog screening test during the 7^th^ follow‐up visit in CARRS‐1 and the 2^nd^ follow‐up of the CARRS‐2 in participants over 50 years old. The Mini‐Cog test is a three‐minute screening instrument that consists of two components: a three‐item recall test for memory and a clock drawing test that yields high sensitivity and specificity for detecting cognitive impairment. In models accounting for age, we tested the association between seven hypothesized modifiable risk factors (education level, tobacco use, alcohol use, sleep duration, fruit and vegetable daily intake, presence of moderate to severe depression, type 2 diabetes, and high blood pressure) and cognitive impairment (Mini‐Cog ≤2 points/5 total points).

**Result:**

A total of 5,711 participants (mean age: 61.0 years; female: 59%) with the Mini‐Cog screening test were included in the analysis. Twenty‐five percent (female: 28.3%; men: 22.7%) of the participants showed indications of cognitive impairment on the Mini‐Cog. We identified an association between higher education levels and lower alcohol intake being linked to a lower risk of cognitive impairment. Additionally, higher tobacco use was directly associated with a greater risk of cognitive impairment. In sex‐stratified analysis, we observed that higher education attainment was protective against cognitive impairment in both men and women. Moreover, tobacco use was specifically associated with cognitive impairment in men, while higher fruit and vegetable intake exerted a protective effect against cognitive impairment only in females (Table).

**Conclusion:**

Over a quarter of the P‐CARRS study participants presented with cognitive impairment. Future longitudinal studies are warranted to explore the association of risk factor control with improving cognitive function, particularly in populations and regions where dementia prevalence is projected to increase, such as in South Asia.